# Distinct colony niches shape associated microbiotas in Neotropical stingless bees

**DOI:** 10.1590/1678-4685-GMB-2026-0023

**Published:** 2026-10-09

**Authors:** Karen Luisa Haag

**Affiliations:** 1 Universidade Federal do Rio Grande do Sul, Departamento de Genética, Programa de Pós-Graduação em Genética e Biologia Molecular, Porto Alegre, RS, Brazil

**Keywords:** Microbiota, stingless bee, microbiome, holobiont

## Abstract

Stingless bees (Meliponini) are an ancient lineage of eusocial insects whose associated microbiomes have been increasingly studied in comparison with those of honey bees. In this review, I synthesize current evidence on microbial communities associated with stingless bees across life stages and colony compartments, emphasizing how patterns of host association differ among distinct biological contexts. I propose that stingless bee microbiomes are not uniformly more environmentally driven than those of honey bees. Instead, strong and evolutionarily conserved filters structure microbial communities associated with larvae and adult guts, whereas greater environmental input is more evident in some colony resources, such as stored food and nest materials. Comparative analyses further indicate that differences between honey bees and stingless bees arise primarily from lineage-specific elaborations in colony biology that created new ecological opportunities for microbial diversification, rather than from fundamental divergence in gut-associated microbiota. Framing stingless bee colonies within a modular framework-where larvae, adult guts, and colony resources represent distinct microbial arenas-provides a coherent way to integrate these contrasting dynamics and to understand how host-microbe associations are maintained, reorganized, and evolve in eusocial systems viewed as modular holobionts.

## Introduction

Stingless bees (Hymenoptera: Apidae, Meliponini) represent one of the most diverse radiations of eusocial insects in the Neotropics, encompassing several hundred species that play a central role in the pollination of native vegetation and a wide range of agricultural crops (Klein et al., 2007; Grüter, 2020). Together with honey bees (Hymenoptera: Apidae, Apini), bumble bees (Hymenoptera: Apidae, Bombini), and orchid bees (Hymenoptera: Apidae, Euglossini), stingless bees belong to the corbiculate bees, a clade characterized by pollen-carrying structures on the hind legs; among these groups, only Apini, Bombini, and Meliponini exhibit eusociality, with complex behavioral repertoires (Michener, 1974).

Over the past decade, corbiculate bees-particularly *Apis mellifera*-have become influential model systems for the study of host-associated microbiomes. Research on honey bees has revealed relatively simple, host-specific, and socially transmitted gut bacterial communities, with demonstrated roles in nutrition, immune regulation, and resistance to pathogens (Martinson et al., 2011; Moran et al., 2012; Powell et al., 2014). These findings have profoundly shaped current conceptual frameworks for insect-microbe interactions and established eusocial bees as key systems for investigating microbiome assembly, stability, and function.

However, extending insights derived from honey bees to other corbiculate lineages requires careful consideration of their evolutionary history, social organization, and colony ecology. Meliponini represent an ancient and deeply divergent lineage within Apidae. Phylogenetic and biogeographic analyses support a Gondwanan origin of stingless bees, followed by extensive diversification driven by vicariance and long-distance dispersal, resulting in their remarkable present-day diversity, particularly in the Neotropics (Rasmussen and Cameron, 2010). Although classical comparative studies proposed independent origins of highly eusocial behavior in honey bees and stingless bees (Winston and Michener, 1977), more recent phylogenomic analyses that control for base compositional bias support a single evolutionary origin of eusociality within corbiculate bees, uniting Apini, Bombini, and Meliponini into a monophyletic eusocial clade with no subsequent reversals to solitary lifestyles (Romiguier et al., 2016).

Importantly, a shared evolutionary origin of eusociality does not imply uniformity in social organization or in the molecular mechanisms underlying social traits. Comparative sociogenomic studies indicate that eusociality has undergone extensive lineage-specific elaboration, resulting in functionally similar but mechanistically distinct solutions across corbiculate lineages (Figure 1 A ). These include modifications in hormonal and neural signaling pathways associated with caste differentiation and social behavior, as well as changes in the development, regulation, and deployment of exocrine glands involved in communication, reproduction, and brood care (Woodard et al., 2011; Trapp et al., 2017; Grüter, 2020; Shell et al., 2021). Genomic resources from stingless bees further corroborate this view, revealing lineage-specific genomic architectures underlying advanced eusociality, including features related to caste differentiation, reproduction, and regulatory complexity (Vojvodic et al., 2013; Kapheim et al., 2015; Opachaloemphan et al., 2018; de Paula Freitas et al., 2020; Waiker et al., 2021; Brenman-Suttner and Zayed, 2026).

**Figure 1:**
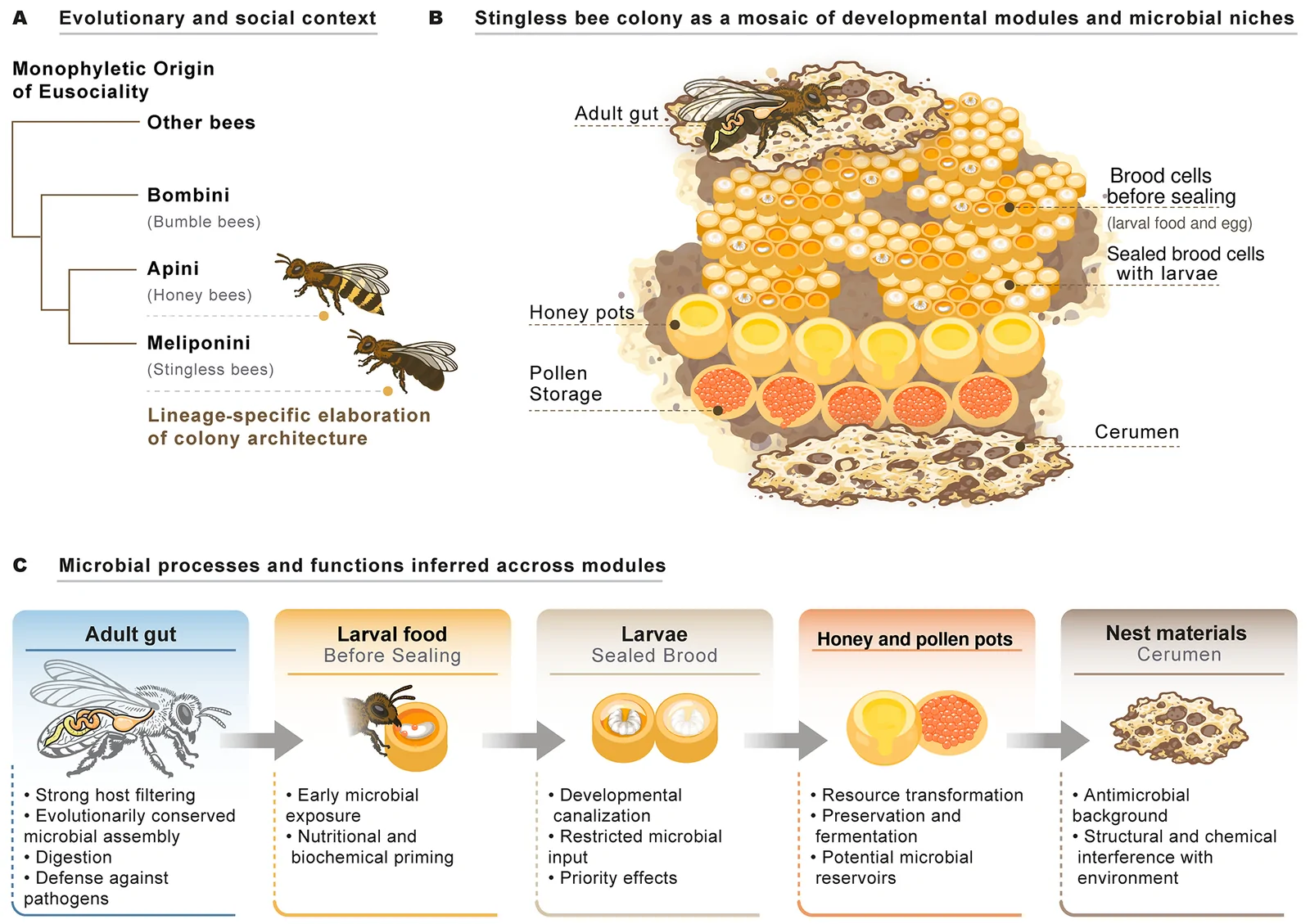
- Modular organization of microbial niches across the stingless bee colony. (A) Evolutionary and social context of corbiculate bees, highlighting the monophyletic origin of eusociality and lineage-specific elaborations of colony architecture in Meliponini. (B) Schematic representation of a stingless bee colony as a mosaic of developmental modules and microbial niches, including larval food before sealing, sealed brood cells with larvae, adult gut, stored pollen and honey, and nest materials such as cerumen. (C) Overview of dominant microbial processes and functions inferred for each module. Adult gut communities are shaped by strong host filtering and evolutionarily conserved assembly processes, whereas larval food, stored resources, and nest materials represent distinct microbial arenas associated with nutritional priming, resource transformation, preservation, and colony-level mediation of host-microbe interactions.

These evolutionary and sociogenomic insights provide a critical framework for understanding microbiome diversity in stingless bees. The social and ecological organization of stingless bee colonies differs profoundly from that of honey bees in ways that are directly relevant to microbial acquisition, transmission, and persistence. Stingless bees construct nests composed of cerumen, a mixture of bee wax plus propolis (or plant resins); brood cells are used only once and subsequently dismantled; and food is stored in discrete honey and pollen pots rather than in multifunctional brood cells (Michener, 1974; Grüter, 2020). Larvae are provisioned with a complete mass of food prior to cell sealing, in contrast to the progressive feeding characteristic of *Apis* (Sakagami, 1982; Grüter, 2020). Communication, recruitment, and colony reproduction further differ in ways that shape patterns of contact, resource flow, and environmental exposure (Kerr and Esch, 1965; Lindauer, 1971; Winston and Michener, 1977; Nieh, 2004; Jarau and Hrncir, 2009; Roubik, 2023).

From a broader perspective, these features suggest that stingless bee colonies constitute spatially structured holobionts, in which microbial communities are distributed across distinct but interconnected developmental modules (Figure 1 B, C ). The holobiont and hologenome concepts emphasize that host-associated microbiotas are integral components of organismal phenotypes, shaped by mixed modes of inheritance, recurrent transmission, and ecological filtering across generations (Bordenstein and Theis, 2015; Haag, 2018; Bovo et al., 2020; Killam et al., 2024; Maurice-Lira et al., 2024). Within this framework, microbiomes contribute to the inheritance of extended phenotypes that influence nutrition, development, immunity, and ecological performance, even in the absence of strict vertical transmission.

Consistent with this view, accumulating evidence indicates that microbial communities associated with stingless bees extend far beyond the adult gut to encompass multiple colony niches, including stored honey and pollen, larval food, brood cells, and nest materials (Michener, 1974; Sakagami, 1982; Rosa et al., 2003; Menezes et al., 2013, 2015; Paludo et al., 2018; Alves et al., 2024; Maurice-Lira et al., 2024; Caesar et al., 2025; Xolalpa-Aroche et al., 2026). These communities comprise both bacteria and fungi, including diverse yeasts that are frequently abundant in stingless bee colonies and stored resources, yet are rare or absent from the gut microbiomes of honey bees or bumble bees (Rosa *et al*., 2003; Kwong et al., 2014, 2017; Cerqueira et al., 2024; Xolalpa-Aroche *et al*., 2026). The strong compartmentalization of resources within stingless bee nests, combined with the extensive use of resin-rich materials and the complete provisioning of brood cells, creates a mosaic of microbial habitats that is likely to influence nutrition, development, and colony health in ways that differ fundamentally from those documented in *Apis*.

In this review, I synthesize current knowledge on the microbiota associated with Neotropical stingless bees within a holobiont framework, with particular emphasis on bacteria and fungi across distinct colony niches. Although stingless bees occur throughout tropical regions worldwide and studies from multiple geographic regions have substantially advanced our understanding of stingless bee microbiomes (Leonhardt and Kaltenpoth, 2014; Kwong et al., 2017; Liu et al., 2023; Mills et al., 2023), the richest body of evidence integrating bee biology with microbial ecology has been generated for Neotropical species (see Table S1 for details). Consequently, they currently provide the most comprehensive framework for understanding how microbial communities are organized across the different ecological and developmental niches of the colony. I therefore begin with the gut microbiota of adult workers-the best-characterized microbial niche in social bees-and then expand to stored resources, larval food, larval stages, brood cells, and nest materials. Across these niches, I discuss patterns of microbial composition, transmission, and functional potential, highlighting available genomic and metabolic evidence as well as major knowledge gaps. By integrating this evidence into a modular holobiont perspective, I propose that the microbiota of Neotropical stingless bees is best understood not as a single gut-associated community, but as a set of interconnected microbial niches shaped by colony organization and developmental transitions.

## Adult gut microbiota of stingless bees

Adult workers are the principal agents connecting the different microbial compartments of the colony. They forage, process and store food, construct and maintain the nest, provision brood cells, and interact continuously with nestmates and developing brood (Grüter, 2020). These behaviors place adult workers at the center of microbial acquisition, transmission, and ecological filtering within the colony. Thus, understanding the adult gut microbiota provides the natural starting point for examining how microbial communities are organized throughout the stingless bee holobiont.

## Composition and core bacterial lineages

The gut microbiota of adult stingless bees has emerged as the best-characterized microbial niche within Meliponini and provides a critical entry point for comparative analyses with other corbiculate bees. Early studies based on culture-dependent approaches already suggested that stingless bee guts harbor structured bacterial communities distinct from those of honey bees, although sharing some higher-level taxonomic affinities (Morais et al., 2013; Marçal, 2017). High-throughput sequencing surveys and genome-resolved analyses have since confirmed that adult workers typically host relatively simple but non-random assemblages dominated by a limited number of bacterial lineages, primarily within the Acetobacteraceae, Lactobacillaceae, and Gammaproteobacteria (Table 1) (Sarton-Lohéac et al., 2023; Haag et al., 2023, 2026; Ramírez-Ahuja et al., 2025; Santini et al., 2026). 

**Table 1 -:** Representative bacterial and fungal taxa reported across major colony niches of Neotropical stingless bees. Rows correspond to colony niches, whereas columns summarize the bee genera investigated. Only taxa explicitly reported as abundant, dominant, recurrent or characteristic within each niche are listed. Empty cells (NA) indicate that comparable data are currently unavailable rather than confirmed absence. Detailed information on host species, study design, detection methods and corresponding references is provided in Supplementary Chart S1.

	Niche	*Frieseomelitta*	*Melipona*	*Plebeia*	*Scaptotrigona*	*Tetragonisca*
Bacteria	Adult gut	*Lactobacillus; Bifidobacterium; Snodgrassella*	*Lactobacillus; Bifidobacterium; Apilactobacillus; Bombella; Floricoccus*	*Lactobacillus; Bifidobacterium; Snodgrassella; Gilliamella; Acetobacter*	*Lactobacillaceae; Bifidobacterium; Bombilactobacillus; Floricoccus; Snodgrassella*	*Snodgrassella; Gilliamella; Lactobacillus; Bifidobacterium*
	Honey	NA	*Rosenbergiella; Acinetobacter; Fructilactobacillus; Saccharibacter; Acetilactobacillus; Nicoliella*	NA	*Acetilactobacillus; Nicoliella*; Lactobacillaceae	NA
	Stored pollen	NA	*Lactococcus; Bacillus; Brevibacillus; Paenibacillus*	NA	*Acetilactobacillus; Nicoliella;* Acetobacteraceae; *Acinetobacter*	NA
	Nest materials (including cerumen)	NA	NA	NA	NA	NA
	Larval food	*Lactobacillus;* Acetobacteraceae	*Lactobacillus;* Acetobacteraceae; *Bacillus; Paenibacillus*	NA	*Acetilactobacillus; Nicoliella; Apilactobacillus; Bombilactobacillus; Bifidobacterium*	*Lactobacillus;* Acetobacteraceae
	Larvae	NA	NA	NA	No stable bacterial genus consistently reported	NA
Fungi	Adult gut	*Starmerella meliponinorum*	*Starmerella; Zygosaccharomyces; Lachancea; Candida; Debaryomyces; Pseudozyma*	NA	Saccharomycetales; *Starmerella; Hanseniaspora; Metschnikowia*	*Starmerella; Wickerhamiella; Hanseniaspora;* variable yeasts
	Honey	*Starmerella meliponinorum*	*Starmerella; Zygosaccharomyces; Metschnikowia;* Saccharomycetales; *Priceomyces*	*Starmerella; Zygosaccharomyces; Wickerhamomyces; Wickerhamiella*	*Starmerella etchellsii; S. bombicola; Zygosaccharomyces; Hanseniaspora*	*Starmerella meliponinorum; Zygosaccharomyces; Hanseniaspora; Wickerhamiella*
	Stored pollen	NA	NA	NA	*Hanseniaspora* and other variable yeasts	*Hanseniaspora opuntiae* and variable yeasts
	Nest materials (including cerumen)	NA	NA	NA	*Candida; Monascus*	NA
	Larval food	*Cladosporium; Saccharomyces*	*Cladosporium; Penicillium; Bipolaris; Didymella;* Saccharomyces	NA	NA	*Erysiphe; Saccharomyces*
	Larvae	NA	NA	NA	*Saccharomycetaceae; Zygosaccharomyces*	NA

Despite superficial similarities to the canonical gut microbiota of *Apis mellifera*, stingless bee gut communities differ markedly in their taxonomic composition and evolutionary histories. In the genus *Melipona*, the adult core gut microbiota is dominated by *Lactobacillus, Bifidobacterium, Apilactobacillus, Bombella,* and *Floricoccus*, whose abundances covary inversely with a set of low-prevalence taxa, indicating a structured and stable core community (Haag et al., 2026). While some bacterial genera-such as *Lactobacillus, Bifidobacterium*, and *Apilactobacillus*-occur in both honey bees and stingless bees, strain-level analyses consistently reveal little to no sharing between hosts. Core honey bee symbionts such as *Snodgrassella alvi* and *Gilliamella apicola*, which dominate the ileum of *Apis* and *Bombus*, are absent or rare in the genus *Melipona* (Kwong et al., 2017; Cerqueira et al., 2021), but are present in the gut of *Scaptotrigona* female workers (Caesar et al., 2025). Importantly, this pattern does not reflect simple geographic divergence: analyses of sympatric *Apis* and *Melipona* colonies have shown that lineage sharing between hosts remains rare even under direct ecological contact (Haag *et al*., 2026). Instead, stingless bees harbor alternative symbiotic lineages that occupy analogous gut compartments but belong to deeply divergent bacterial clades (Sarton-Lohéac et al., 2023), in some cases likely representing undescribed species. In stingless bees, adult gut microbiomes may also vary among subcastes; in *Scaptotrigona* spp., nurse workers harbor a core microbiota dominated by *Acetilactobacillus, Bombilactobacillus*, and *Bifidobacterium*, while foragers and soldiers exhibit more diverse communities and reduced *Acetilactobacillus*, consistent with task-related rather than age-related differences (Caesar *et al*., 2025).

Genome-resolved analyses have provided strong evidence that several of these stingless bee-associated bacteria represent ancient and host-specialized symbionts. Phylogenomic reconstructions reveal deep divergence among gut bacterial lineages associated with different Meliponini genera, consistent with long-term host association punctuated by extinction and replacement events in specific clades (Cerqueira et al., 2021; Sarton-Lohéac et al., 2023). These patterns contrast with the more conserved symbiont assemblages observed across *Apis* species and highlight the role of host lineage history in shaping gut microbiome composition.

## Spatial organization and gut compartmentalization

As in other corbiculate bees, the adult stingless bee gut is anatomically divided into functionally distinct compartments-the crop, midgut, and hindgut-each imposing different physicochemical constraints on microbial colonization. Recent microscopy-based and amplicon sequencing studies indicate that dominant bacterial taxa exhibit consistent spatial localization patterns along the gut, suggesting niche partitioning driven by gradients in oxygen availability, pH, and substrate composition (Santini et al., 2026). As in honey bees and bumble bees (Martinson et al., 2011, 2012; Engel et al., 2012), the gut bacterial communities of stingless bees are spatially structured along the alimentary tract, with distinct assemblages associated with the crop and hindgut. Acetobacteraceae-related symbionts are frequently enriched in the crop and anterior midgut, environments characterized by fluctuating sugar concentrations and intermittent oxygen exposure, whereas Lactobacillaceae and other fermentative bacteria tend to dominate the hindgut, where anaerobic conditions and host-derived substrates prevail (Santini *et al*., 2026). Within the hindgut, available evidence suggests further differentiation between the ileum and rectum, paralleling the compartmentalization described for honey bees, in which the ileum is dominated by specialized biofilm-forming symbionts such as *Snodgrassella* and *Gilliamella*, while the rectum harbors dense fermentative communities dominated by *Bifidobacterium* and lactobacilli (Martinson *et al*., 2011; Engel *et al*., 2012). Notably, although this spatial organization is functionally similar across corbiculate bees, it is achieved through taxonomically distinct microbial lineages in stingless bees, reflecting independent evolutionary trajectories of host-microbe associations rather than simple conservation of symbiont taxa (Haag et al., 2026).

## Functional potential and metabolic roles

Although functional characterization of stingless bee gut symbionts remains incomplete, genomic and metabolic inference suggests that bee gut bacteria contribute primarily to carbohydrate metabolism and fermentation (Engel et al., 2012; Kwong and Moran, 2016). Many stingless bee dominant gut-associated lineages possess genes involved in the breakdown of simple and complex sugars derived from nectar and pollen, as well as pathways for the production of organic acids such as acetate and lactate (Sarton-Lohéac et al., 2023; Caesar et al., 2025). These metabolic activities are likely to influence gut physicochemistry, inhibit opportunistic pathogens, and modulate host nutrient availability.

Comparative studies in honey bees have shown that gut symbionts occupy partially overlapping metabolic niches, with functional redundancy distributed across taxa rather than concentrated in single species (Bonilla-Rosso and Engel, 2018). A similar principle may apply in stingless bees, where functionally analogous roles appear to be fulfilled by distinct bacterial lineages. However, whether metabolic specialization and cross-feeding interactions in Meliponini reach the same level of integration described for *Apis* remains an open question.

Experimental evidence further indicates that the adult gut microbiota of stingless bees is sensitive to dietary inputs. Manipulative studies in managed colonies demonstrate that supplemental feeding can alter the relative abundance of core bacterial taxa without necessarily disrupting overall community structure or host physiological traits, such as fat body size (Haag et al., 2025). These findings suggest a degree of functional resilience and host-mediated dietary control over gut community composition.

Overall, the adult gut microbiota of stingless bees comprises structured microbial assemblages shaped by deep host divergence, lineage-specific ecological pressures, and social transmission. Understanding how these communities interact with host physiology, diet, and colony-level processes will be essential for integrating stingless bees into broader comparative frameworks of eusocial insect microbiomes.

## Colony-associated microbiota

Stored resources constitute a central component of stingless bee colony organization and represent one of the most distinctive ecological features separating Meliponini from honey bees. Unlike *Apis mellifera*, which stores pollen and nectar within multifunctional comb cells, stingless bees store honey and pollen in separate cerumen pots or sites within the colony, spatially segregated from the brood area and frequently closed (Michener, 1974; Sakagami, 1982). These structures generate chemically and physically heterogeneous microhabitats that impose strong selective pressures on microbial colonization and persistence.

## Cerumen and resins

Stingless bee brood cells and nest materials form chemically complex microhabitats composed primarily of wax and resinous materials (cerumen), frequently enriched with propolis and, in some species, soil-derived particles (Shanahan and Spivak, 2021; Vit et al., 2024). These materials exhibit antimicrobial properties and are known to influence microbial growth and persistence within nests (Michener, 1974; Roubik, 2006, 2024). Culture-based and molecular studies have demonstrated that cerumen and brood cell-associated substrates harbor distinct microbial assemblages, including bacteria and fungi that differ from those found in adult guts and stored food resources (Rosa et al., 2003; Menezes et al., 2013). Although the functional roles of these communities remain poorly characterized, existing evidence indicates that nest materials contribute to shaping the spatial organization of microbes within stingless bee colonies and may act as environmental filters affecting microbial exposure during brood development.

## Honey and pollen stores

Honey and pollen stores in stingless bee nests are characterized by high sugar concentrations, variable water activity, acidic pH, frequent incorporation of plant resins and waxes, and ongoing microbial fermentation during resource maturation (Shanahan and Spivak, 2021; Pimentel et al., 2022; da Silva et al., 2024). Together, these features create environments that differ markedly from both the adult gut and the brood cells, favoring microorganisms capable of tolerating osmotic stress, acidity, and exposure to antimicrobial compounds derived from plant resins (Roubik, 2006). As a consequence, the microbiota associated with stored resources is compositionally and functionally distinct from that of adult workers (Leonhardt and Kaltenpoth, 2014). Consistent with this ecological filtering, recent strain-resolved metagenomic analyses in *Scaptotrigona* spp. show that microbial communities associated with honey and pollen stores are not only compositionally distinct, but also harbor largely non-overlapping bacterial strains relative to other nest compartments. Despite partial overlap at higher taxonomic levels, strain sharing among hive components is rare, indicating strong spatial segregation of microbial populations within the nest (Caesar et al., 2025).

Comparative studies indicate that pollen and honey pots constitute partially independent microbial niches within stingless bee colonies, with substrate type emerging as a major determinant of community structure (Rosa et al., 2003). Culture-based surveys recovered diverse cultivable bacteria and yeasts from honey and pollen stores. Studies targeting fructophilic lactic acid bacteria subsequently combined selective isolation with 16S rRNA gene sequencing to identify *Fructilactobacillus* species associated with stingless bee honey, further confirming that these resources harbor metabolically active microbial communities (Beux et al., 2022; Andrade-Velásquez et al., 2023). Recent genome-resolved metagenomic analyses indicate that honey stores are dominated by a restricted set of highly specialized Lactobacillaceae, including *Acetilactobacillus*-like and *Nicoliella*-like lineages, whereas pollen stores harbor more diverse fermentative microbial assemblages. These findings suggest that honey represents a highly selective microbial environment that supports specialized bacterial communities across multiple stingless bee taxa (Caesar et al., 2025; Xolalpa-Aroche et al., 2026). 

## Community composition: bacteria, yeasts, and filamentous fungi

Culture-based, amplicon sequencing, and more recently genome-resolved metagenomic studies consistently demonstrate that fungi-particularly yeasts-are a prominent and persistent component of the microbiota associated with stored resources in stingless bee colonies (Table 1). Yeast taxa frequently reported include members of *Candida*, *Starmerella*, *Wickerhamiella*, *Zygosaccharomyces*, and related genera, many of which are well known for their ability to grow under high sugar concentrations and low pH (Rosa et al., 2003). In several stingless bee species, these yeasts appear to be recurrent and sometimes species-specific, suggesting stable associations rather than incidental contamination (Rosa *et al*., 2003).

Bacterial communities in stored resources are typically dominated by members of the Lactobacillaceae (including *Acetilactobacillus, Bombilactobacillus,* and related genera), Acetobacteraceae, Bacillaceae, Actinobacteria, and, in pollen-associated niches, Proteobacteria such as *Acinetobacter* (Rosa et al., 2003; Morais et al., 2013; Santos et al., 2023; Alves et al., 2024; Caesar et al., 2025; Xolalpa-Aroche et al., 2026). Importantly, the relative abundance and composition of these bacterial assemblages vary substantially among host species, colonies, and geographic regions, indicating that both host-related factors and local environmental conditions contribute to shaping these communities (Morais *et al*., 2013; Kueneman et al., 2023). Collectively, these studies suggest that the microbiota of stored resources is not simply a reflection of the adult gut microbiota, but rather represents specialized microbial communities shaped by the physicochemical properties of honey and pollen and by microbial succession during resource maturation.

Honey yeast composition varies markedly among stingless bee species. In a survey of 17 Meliponini, *Starmerella* sp. predominated in several *Melipona* honeys, *Starmerella bombicola* was restricted to *Scaptotrigona*, *Zygosaccharomyces mellis* dominated *Melipona quadrifasciata*, and *Wickerhamomyces sydowiorum* was particularly abundant in *Plebeia saiqui*, revealing a strong host-species signature despite shared environmental conditions (Echeverrigaray et al., 2021). Filamentous fungi such as *Penicillium* and *Talaromyces* are also frequently detected in pollen pots, although their ecological roles remain less well understood. Recent studies suggest that these fungi can contribute to the degradation of pollen walls, the production of secondary metabolites, or interactions with bacterial and yeast communities that influence resource quality and stability (Santos et al., 2023; Roque et al., 2024; Caesar et al., 2025). 

## Functional roles in resource transformation and preservation

Accumulating evidence indicates that microbial activity within stored resources is an integral component of resource maturation, contributing to the biochemical transformation and preservation of pollen- and nectar-derived substrates (Menezes et al., 2013; Anderson et al., 2014; Maurice-Lira et al., 2024). Fermentation processes mediated by bacteria and yeasts can alter sugar composition, reduce pH, and generate organic acids and other metabolites that inhibit the growth of spoilage organisms and potential pathogens (Morais et al., 2013). These transformations may enhance the nutritional value, digestibility, or storability of pollen and honey, thereby extending the temporal availability of food resources within the colony.

In this respect, stored resources in stingless bees share functional parallels with bee bread in honey bees and pollen provisions in bumble bees, where microbial succession and metabolic activity play important roles in food preservation (Anderson et al., 2013; Dharampal et al., 2020; Disayathanoowat et al., 2020). However, the prominence and diversity of fungi in stingless bee stored resources distinguish Meliponini from *Apis*, where fungal components are generally less abundant and more transient. Recent metagenomic analyses provide direct functional support for these roles. In *Scaptotrigona*, protein-based annotations reveal that honey-associated microbiomes are enriched in functions related to antimicrobial activity and spoilage prevention, whereas pollen-associated communities display a broader functional repertoire consistent with fermentative metabolism and resource transformation (Caesar et al., 2025). Likewise, genome-resolved metagenomics of pot honey from *Melipona beecheii* and *Scaptotrigona mexicana* revealed highly specialized Lactobacillaceae lineages that appear to be recurrent components of stingless bee honey, further supporting the view that microbial communities actively contribute to the maturation and stability of stored resources (Xolalpa-Aroche et al., 2026).

Experimental and comparative data further suggest that microbial communities in stored resources may contribute to colony-level defense. Organic acids, ethanol, and other fermentation-derived metabolites can suppress the growth of opportunistic microbes, while resin-associated compounds incorporated into pots may act synergistically with microbial metabolites to stabilize stored food (Roubik, 2006; Menezes et al., 2013).

## Development-associated microbiota

Larval food represents a pivotal interface between stored resources, microbial communities, and host development in stingless bee colonies. Unlike honey bees, in which larvae are progressively fed, stingless bee larvae receive a complete mass of food prior to cell sealing (Figure 2) (Grüter, 2020). This mass provisioning creates a closed microenvironment in which microbial communities can develop, persist, and interact with the larva throughout its entire immature phase (Michener, 1974; Sakagami, 1982).

**Figure 2:**
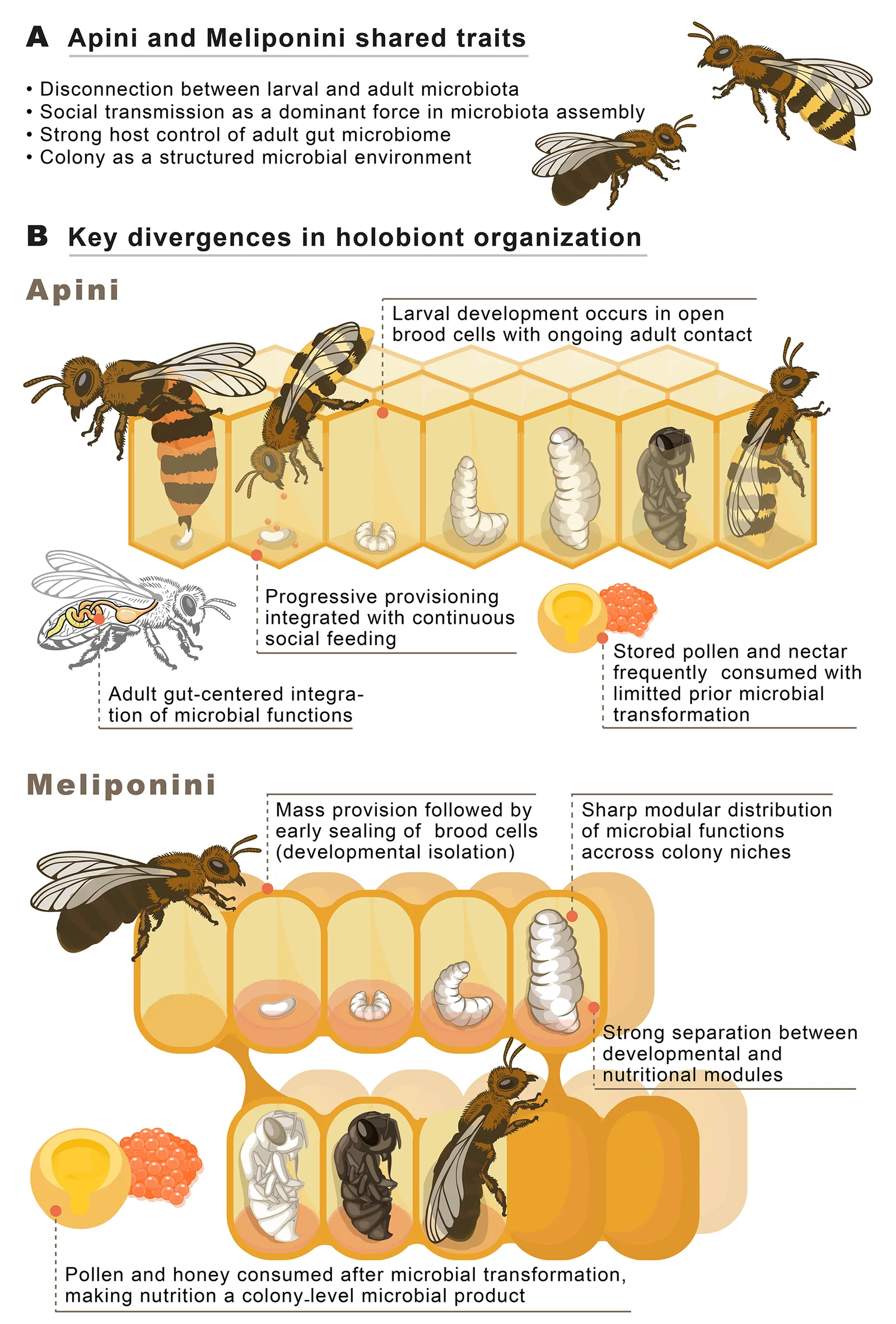
- Shared features and key divergences in holobiont organization between Apini and Meliponini. (A) Traits shared by honey bees (Apini) and stingless bees (Meliponini), including the decoupling between larval and adult microbiota, the central role of social transmission in microbiota assembly, strong host control of the adult gut microbiota, and the colony functioning as a structured microbial environment. (B) Major divergences in holobiont organization linked to differences in colony architecture and provisioning strategies. In Apini, progressive provisioning and continuous adult-larva contact promote a gut-centered integration of microbial functions. In Meliponini, mass provisioning followed by early brood cell sealing leads to developmental isolation and a sharp modular distribution of microbial functions across colony niches, with nutrition emerging as a colony-level microbial product shaped through stored and transformed resources.

## Composition and origin of larval food microbiota

Studies across multiple stingless bee species consistently show that larval food harbors complex microbial assemblages composed of bacteria, yeasts, and filamentous fungi (Table 1). These communities partially overlap with those found in stored pollen and honey, supporting the view that larval food microbiota is largely derived from resource-associated microbes introduced during provisioning (Rosa et al., 2003; Morais et al., 2013). However, larval food also exhibits distinct microbial profiles, suggesting that selective processes operate during or after provisioning.

Bacterial communities in larval food frequently include members of Lactobacillaceae, Acetobacteraceae, Bacillaceae, and Actinobacteria, taxa commonly associated with fermentative metabolism and acid production. Fungal components are often prominent and may include both yeasts and filamentous fungi, some of which recur across colonies and host species (Menezes et al., 2013; Santos et al., 2023). In *Scaptotrigona*, analyses of hive compartments show that larval food is characterized by a distinct assemblage dominated by *Acetilactobacillus, Bombilactobacillus*, and *Bifidobacterium*. Larval food also differs from pollen and honey stores in its fungal composition, showing lower diversity and reduced representation of the osmotolerant yeast lineages that dominate stored resources, consistent with stronger ecological filtering during larval provisioning (Caesar et al., 2025). This pattern may also be consistent with microbial priming, whereby early-established bacterial lineages in larval provisions limit subsequent colonization by fungi that are abundant in pollen and honey stores, thereby generating priority effects during community assembly (Figure 1 C ). The relative abundance and diversity of microbial groups vary among stingless bee species, indicating strong host-specific and ecological effects.

## Functional roles during larval development

Accumulating evidence indicates that microorganisms present in larval food contribute actively to larval development (Menezes et al., 2013; Paludo et al., 2018; Paludo *et al*., 2019). Microbial metabolism can modify the physicochemical properties of larval food, including pH, redox conditions, and nutrient composition, potentially enhancing digestibility and nutrient availability. Fermentative activity may also generate organic acids and other metabolites that inhibit opportunistic microbes, stabilizing the larval environment during development (Santos et al., 2022). Functional profiling of larval food microbiomes further reveals enrichment in genes associated with carbohydrate metabolism, organic acid production, and biosynthesis of vitamins and cofactors, supporting the view that these communities actively transform the larval substrate (Caesar et al., 2025).

In some stingless bee lineages, the relationship between larvae and their associated microbiota reaches the level of functional dependency. Experimental studies demonstrate that larvae of certain species require microbially derived compounds to successfully complete metamorphosis, indicating obligate or near-obligate symbioses mediated through larval food (Menezes *et al*., 2015; Paludo et al., 2018, 2019; de Paula et al., 2023). In these systems, specific yeasts cultivated within the larval food-most notably *Zygosaccharomyces* spp.-produce sterols and other metabolites essential for pupation, directly linking microbial metabolism to host developmental pathways. While obligate sterol provisioning has been demonstrated for *Zygosaccharomyces* in some stingless bee lineages, other recurrent yeasts such as *Starmerella* are instead thought to contribute primarily through fermentation, lipid metabolism, and antimicrobial compound production, without evidence for direct sterol-dependent developmental roles.

## Host-microbe interactions and caste-related effects

Larval food microbiota may also modulate developmental outcomes among castes without overriding the mechanisms underlying caste determination. In the genus *Melipona*, caste differentiation is now understood to result from the interaction between genetic, nutritional, endocrine, and epigenetic factors (Grüter, 2020). The classical two-locus model proposed by Kerr established the existence of a genetic component influencing caste fate (Kerr, 1950; Maciel-Silva and Kerr, 1991), while subsequent studies demonstrated that larval nutrition interacts with hormonal pathways and epigenetic mechanisms-including DNA methylation and histone modifications-to regulate developmental trajectories (Hartfelder et al., 2006; Cardoso-Júnior et al., 2017; Luna-Lucena et al., 2019; Schumann et al., 2019; Borges et al., 2021; Pieplow et al., 2026).

Within this integrative framework, variation in the microbial composition or metabolic activity of larval food is unlikely to determine caste fate directly, but may influence the nutritional and biochemical environment in which genetic, endocrine, and epigenetic regulatory pathways operate. Consequently, microorganisms associated with larval food may affect larval growth, survival, physiological condition, and the successful execution of caste-specific developmental programs. Recent studies suggest that differences in fungal diversity and abundance within larval food are associated with queen development and larval survival in some Meliponini species (de Queiroz et al., 2025). Rather than acting as primary determinants of caste differentiation, these microbial communities are therefore more likely to modulate developmental performance by interacting with the broader network of mechanisms governing caste development.

Although the molecular mechanisms underlying these effects remain poorly resolved, microbial contributions to sterol metabolism, hormone precursors, or signaling molecules provide plausible links between larval food microbiota and host developmental gene networks. These interactions highlight larval food as a key context in which microbial functions intersect directly with host genetics and physiology.

## Larval microbiota in stingless bees

The larval microbiota of stingless bees constitutes a distinct biological entity from both larval food microbiota and adult gut communities. Although larvae develop immersed in microbially rich provisions, only a subset of these microorganisms successfully colonizes larval surfaces and the gut. Consequently, larval-associated communities are consistently less diverse than larval food and stored resources, reflecting strong host- and developmentally mediated filtering rather than passive carryover from the surrounding food matrix (Vojvodic et al., 2013; Kowallik and Mikheyev, 2021; Santos et al., 2023). Available studies indicate that these communities are typically dominated by Lactobacillaceae (Table 1), together with members of the Proteobacteria and Actinobacteria, although their relative abundances vary among host species, larval instars, and colony conditions (Ortiz, 2019; Gavazzoni et al., 2023).

Larval-associated microbial communities also change throughout development. Following brood provisioning, larvae are initially exposed to a diverse assemblage of food- and nest-associated microorganisms, but community membership progressively narrows as development proceeds, consistent with continued ontogenetic filtering and host-mediated selection (Ortiz, 2019; Gavazzoni et al., 2023). This process culminates during metamorphosis, when most larval-associated microorganisms are lost, effectively decoupling larval and adult microbiomes (Figure 2 A ) (Kowallik and Mikheyev, 2021).

## Toward an integrated framework for colony-associated microbiomes

The studies reviewed here collectively indicate that microbial communities associated with stingless bees cannot be fully understood by considering individual colony compartments in isolation. Instead, adult guts, stored resources, larval food, brood cells, developing larvae and nest materials form interconnected microbial systems linked by recurrent transmission, ecological filtering and colony-level niche construction. Within a holobiont framework, the colony therefore emerges as the ecological context in which host and microbial components are repeatedly assembled and integrated across developmental stages.

## Recurrent microbial transmission maintains colony-level microbial continuity

One of the central questions emerging from studies of social bee microbiomes is how microbial continuity is maintained across generations despite the profound developmental discontinuities imposed by complete metamorphosis. Unlike vertically inherited intracellular symbionts, most microorganisms associated with eusocial bees occupy extracellular habitats and are acquired repeatedly throughout the life cycle. Consequently, the persistence of characteristic microbial communities cannot be explained simply by inheritance from parent to offspring but instead depends on recurrent ecological and social transmission operating at the colony level.

Complete metamorphosis represents one of the major bottlenecks for microbial persistence in holometabolous insects. During pupation, extensive tissue remodeling results in the loss of much of the larval-associated microbiota, effectively decoupling larval and adult microbial communities (Vojvodic et al., 2013; Hroncova et al., 2015; Kowallik and Mikheyev, 2021). Consequently, microorganisms associated with larval food or larval tissues generally do not constitute the primary inoculum of the adult gut microbiota. Instead, newly emerged workers reconstruct their characteristic microbial communities after adult emergence through repeated interactions with nestmates and the colony environment.

In corbiculate bees, this reconstruction is achieved through multiple, partially overlapping transmission routes. Social interactions among nestmates, contact with nest materials, consumption of stored food, trophallaxis, and repeated exposure to the colony environment all contribute to the acquisition and maintenance of characteristic microbial communities (Powell et al., 2014; Onchuru et al., 2018; Drew et al., 2021). Rather than representing isolated transmission events, these processes operate continuously throughout adult life, generating recurrent opportunities for microbial exchange while simultaneously exposing microorganisms to successive ecological filters within different colony compartments (Graystock et al., 2017; McFrederick et al., 2017; Motta and Moran, 2024).

Current evidence indicates that stingless bees follow this general pattern of recurrent microbial transmission. Adult workers continuously move microorganisms among flowers, stored resources, larval food, brood cells and nest materials while foraging, processing food and provisioning brood (de Paula et al., 2023; Cerqueira et al., 2024). Consequently, colony-level microbial continuity is maintained not through the persistence of identical microbial assemblages across all life stages, but through the repeated reconstruction of functionally appropriate communities. The relative strength of ecological filters is likely to differ among colony compartments and host species (Graystock et al., 2017), potentially contributing to the remarkable diversity observed across Meliponini.

This perspective shifts the focus from transmission among individuals to transmission among colony compartments. Rather than viewing the adult gut as the primary reservoir of the microbiome, the colony itself emerges as the principal framework within which microorganisms are acquired, filtered, redistributed and reassembled across successive generations (Rokop et al., 2015; Anderson and Copeland, 2024; Cerqueira et al., 2024). Adult workers occupy a pivotal position in this process, repeatedly linking environmental microbial reservoirs with the developmental and ecological niches that together sustain colony function. This colony-level continuity provides the conceptual basis for interpreting the distinct microbial communities associated with stored resources, larval food, brood cells, developing larvae and adult guts as interconnected components of a single microbial system.

## Neotropical stingless bees as a model for comparative studies of bee microbiomes

The growing recognition that microbial communities occupy multiple interconnected niches within eusocial bee colonies opens new opportunities for comparative studies across corbiculate bees. Rather than focusing exclusively on the adult gut, this broader perspective allows colony-associated microbiomes to be interpreted in light of the ecological and developmental contexts in which they are assembled. Within this comparative framework, Neotropical stingless bees occupy a particularly informative position. They comprise the largest evolutionary radiation of Meliponini, accounting for approximately 77% of the global diversity with over 420 described species, compared to only 16% in the Indo-Malayan/Australasian region and 7% in the Afrotropics (Grüter, 2020). This geographical bias encompasses extraordinary diversity in nest architecture, colony organization, resource storage strategies and interactions with plant-derived materials. Together, these characteristics generate a wide range of ecological contexts in which host-associated microbial communities assemble and persist.

Importantly, this diversity should not necessarily be interpreted as evidence that all microbial communities associated with stingless bees are intrinsically more diverse or more environmentally derived than those of other eusocial bees. Rather, different colony compartments are expected to experience different ecological constraints and therefore should be compared with equivalent niches in other bee lineages. Indeed, the adult gut illustrates this point well. Comparative analyses performed within the same geographical locality indicate that *Melipona* and *Apis mellifera* harbor similarly structured gut bacterial communities despite their distinct colony biology (Haag et al., 2026). This observation suggests that strong host-mediated filtering remains a conserved feature of gut community assembly across corbiculate bees, whereas greater variation may emerge in other colony-associated habitats.

Viewed in this way, the exceptional diversity of Neotropical stingless bees provides an opportunity to investigate how evolutionary changes in colony biology influence the organization of colony-associated microbiomes. Variation in nest architecture (Roubik, 2006), the use of chemically diverse resins (Leonhardt, 2017; Shanahan and Spivak, 2021), the incorporation of mineral soil by many taxa (Chuttong et al., 2023; Vit et al., 2024), and differences in brood provisioning, food storage and fungal associations (Paludo et al., 2019; de Paula et al., 2023) collectively create an unparalleled comparative framework for examining how host behavior and colony ecology shape microbial communities across multiple biological compartments.

Recent conceptual advances in honey bees have similarly emphasized the importance of considering colony-associated microbial habitats beyond the adult gut (Anderson et al., 2011; Anderson *et al*., 2013; Rokop et al., 2015; Anderson and Copeland, 2024). Together, these studies provide the foundation for a broader comparative perspective in which colony biology, rather than individual microbial niches alone, becomes the central unit for understanding host-microbe interactions in eusocial bees.

## Conclusion and future perspectives

The holobiont concept has profoundly influenced current thinking on host-microbe interactions by emphasizing that host phenotypes emerge from the integration of host and microbial components (Bordenstein and Theis, 2015; Rosenberg and Zilber-Rosenberg, 2016). The evidence reviewed here suggests that, in eusocial bees, this integration extends beyond the individual organism to encompass multiple interconnected microbial habitats generated by colony biology. Neotropical stingless bees greatly expand the comparative landscape available for investigating how colony organization, developmental biology and ecological diversification influence microbiome assembly. Their exceptional evolutionary radiation provides an opportunity to examine how different solutions to eusocial life generate alternative configurations of colony-associated microbial systems.

Future studies integrating comparative microbiomics, microbial ecology, metabolomics and experimental manipulation across multiple stingless bee lineages will be essential to determine which features of colony-associated microbiomes represent conserved properties of eusociality and which reflect lineage-specific evolutionary innovations. Such comparative approaches may ultimately reveal how the diversification of colony biology has shaped the evolution of host-microbe interactions in social insects.

## Data Availability

This is a review article, and no new datasets were generated or analyzed during its preparation. All information discussed is available in the published literature cited in the manuscript.

## Supplementary material

The following online material is available for this article:

Table S1 - Microbial taxa reported across life stages and colony-associated niches of Neotropical stingless bees.
