# Supplementary material for: Distinct colony niches shape associated microbiotas in Neotropical stingless bees
Source: Genet Mol Biol. 2026 Oct 9;49(Suppl 1):e20260023. doi: 10.1590/1678-4685-GMB-2026-0023 (PMC13651872; doi:10.1590/1678-4685-GMB-2026-0023)
Supplement: Supplementary file 1 [file 1415-4757-GMB-49-s1-e20260023-s1.pdf]

*Genetics and Molecular Biology*
https://doi.org/10.1590/1678-4685-GMB-2026-0023

# Supplementary Material to "Distinct colony niches shape associated microbiotas in Neotropical stingless bees"

**Table S1 -** Microbial taxa reported across life stages and colony-associated niches of Neotropical stingless bees.

| **Study** | **Year** | **Bee genus** | **Bee species** | **Life stage / material** | **Specific niche** | **Bacteria reported** | **Fungi reported** |
|---|---|---|---|---|---|---|---|
| Rosa et al. | 2003 | *Tetragonisca* | *T. angustula* | Adult bee surface-associated | Adult bees | Not assessed | *Starmerella meliponinorum* dominant; *Aureobasidium pullulans; Candida etchellsii; Candida versatilis*-like; *Candida* sp.; *Cryptococcus* albidus-like; *Cryptococcus* spp.; *Debaryomyces hansenii; Issatchenkia scutulata* var. *exigua*-like; *Pseudozyma antarctica; Rhodotorula* spp. |
| Rosa et al. | 2003 | *Tetragonisca* | *T. angustula* | Colony resource | Honey | Not assessed | *Starmerella meliponinorum* in all honey samples; *Zygosaccharomyces bisporus*-like |
| Rosa et al. | 2003 | *Tetragonisca* | *T. angustula* | Colony resource | Pollen provisions | Not assessed | *Starmerella meliponinorum* |
| Rosa et al. | 2003 | *Tetragonisca* | *T. angustula* | Nest material | Garbage pellets | Not assessed | *Starmerella meliponinorum; Candida etchellsii; Candida* sp.; *Cryptococcus* spp.; *Rhodotorula* spp. |
| Rosa et al. | 2003 | *Tetragonisca* | *T. angustula* | Nest material | Propolis | Not assessed | *Starmerella meliponinorum; Debaryomyces hansenii* |
| Rosa et al. | 2003 | *Melipona* | *M. quadrifasciata* | Adult bee surface-associated | Adult bees | Not assessed | *Candida apicola* complex dominant; *Debaryomyces hansenii; Pseudozyma antarctica; Aureobasidium pullulans; Candida catenulata; Candida floricola; Cryptococcus laurentii; Clavispora macerans; Kodamaea ohmeri; Rhodotorula* sp.; *Starmerella meliponinorum; Zygosaccharomyces* sp. |
| Rosa et al. | 2003 | *Melipona* | *M. quadrifasciata* | Colony resource | Honey | Not assessed | *Candida apicola* complex; *Starmerella meliponinorum; Kodamaea ohmeri* |

| Study | Year | Bee genus | Bee species | Life stage / material | Specific niche | Bacteria reported | Fungi reported |
|---|---|---|---|---|---|---|---|
| Rosa et al. | 2003 | *Melipona* | *M. quadrifasciata* | Nest material | Garbage pellets | Not assessed | *Candida apicola* complex; *Debaryomyces hansenii; Kodamaea ohmeri* |
| Rosa et al. | 2003 | *Frieseomelitta* | *F. varia* | Adult bee surface-associated | Adult bees | Not assessed | *Starmerella meliponinorum* in all samples; *Aureobasidium pullulans; Candida versatilis*-like; *Kloeckera* sp. |
| Rosa et al. | 2003 | *Frieseomelitta* | *F. varia* | Colony resource | Honey | Not assessed | *Starmerella meliponinorum* |
| Rosa et al. | 2003 | *Frieseomelitta* | *F. varia* | Nest material | Garbage pellets | Not assessed | *Candida versatilis*-like; *Zygosaccharomyces* sp. |
| Marçal | 2017 | *Melipona* | *M. seminigra* | Adult worker | Adult gut: crop, midgut and hindgut | *Lactobacillus* (dominant); *Bifidobacterium; Snodgrassella; Pectinatus; Rosenbergiella;* rare *Lactococcus, Tatumella, Anaplasma, Fructobacillus* and *Bombella; Acetobacteraceae* | Not assessed |
| Kwong et al. | 2017 | *Plebeia* | *P. droryana* | Adult worker | Adult gut | *Snodgrassella; Gilliamella; Lactobacillus* Firm-4*; Lactobacillus* Firm-5*; Bifidobacterium; Acetobacter*-like OTU | Not assessed |
| Marçal | 2017 | *Melipona* | *M. seminigra* | Colony resource | Corbicular pollen | Highly diverse environmental and bee-associated bacteria; *Bacillus*, *Dysgonomonas* and some *Enterococcus* phylotypes exclusive; *Weissella, Lactobacillus* and *Enterococcus* comparatively abundant | Not assessed |
| Marçal | 2017 | *Melipona* | *M. seminigra* | Colony resource | Stored pollen in open pots | *Lactococcus* prominent in sequencing data; culturable *Brevibacillus, Bacillus* and *Paenibacillus* dominant; *Staphylococcus* and *Aneurinibacillus* also recovered | Not assessed |
| Marçal | 2017 | *Melipona* | *M. seminigra* | Colony resource | Stored pollen in closed pots | *Lactococcus* prominent in sequencing data; culturable *Brevibacillus, Bacillus* and *Paenibacillus* dominant | Not assessed |
| Marçal | 2017 | *Melipona* | *M. seminigra* | Larval provision | Larval food | Sequencing data dominated by *Lactococcus;* culture collection dominated by *Brevibacillus, Bacillus* and *Paenibacillus; Staphylococcus,* | Not assessed |

| Study | Year | Bee genus | Bee species | Life stage / material | Specific niche | Bacteria reported | Fungi reported |
|---|---|---|---|---|---|---|---|
| | | | | | | *Aneurinibacillus* and *Convivina* also detected | |
| Paludo et al. | 2019 | *Scaptotrigona* | *S. depilis* | Brood provision | Brood cells | Not assessed | *Zygosaccharomyces* sp.; *Candida* sp.; *Monascus ruber* |
| Paludo et al. | 2019 | *Scaptotrigona* | *S. depilis* | Nest material | Brood-cell cerumen | Not assessed | *Candida* sp.; *Monascus ruber* |
| Cerqueira et al. | 2021 | *Melipona* | Multiple species from four Brazilian biomes | Adult worker | Adult gut | *Lactobacillus;* Bifidobacteriaceae; Acetobacteraceae; *Floricoccus; Snodgrassella* and *Gilliamella* extremely rare or absent | *Starmerella; Lachancea; Zygosaccharomyces; Wickerhamomyces; Wickerhamiella; Metschnikowia; Candida* |
| Cerqueira et al. | 2021 | *Tetragonisca* | *T. angustula* | Adult worker | Adult gut | *Snodgrassella; Gilliamella; Lactobacillus;* Bifidobacteriaceae; other core corbiculate bacteria | Variable yeasts; lower *Starmerella* prevalence than in *Melipona* |
| Echeverrigaray et al. | 2021 | *Melipona* | *M. bicolor, M. fasciculata, M. marginata, M. mondury, M. quadrifasciata, M. scutellaris, M. seminigra* | Colony resource | Honey | Not assessed | *Starmerella* sp. prevalent in several species; *S. apicola* in *M. fasciculata* and *M. scutellaris* and at lower incidence in *M. marginata* and *M. mondury; Zygosaccharomyces mellis* dominant in *M. quadrifasciata; Candida patagonica* in *M. fasciculata; Debaryomyces hansenii* in *M. seminigra* |
| Echeverrigaray et al. | 2021 | *Plebeia* | *P. droryana, P. emerina, P. nigriceps, P. remota, P. saiqui* | Colony resource | Honey | Not assessed | *Starmerella* sp.; *S. apicola* in *P. emerina* and *P. saiqui*; *Zygosaccharomyces mellis* and *Z. rouxii; Wickerhamomyces sydowiorum* in *P. saiqui; Wickerhamiella versatilis* sole species in *P. nigriceps* |
| Echeverrigaray et al. | 2021 | *Scaptotrigona* | *S. bipunctata, S. depilis, S. tubiba* | Colony resource | Honey | Not assessed | *Starmerella* sp.; *S. etchellsii* restricted to the three *Scaptotrigona* species; *S. bombicola* in all *S. bipunctata* and *S. depilis* samples; *Zygosaccharomyces mellis* in *S. depilis* |
| Echeverrigaray et al. | 2021 | *Tetragonisca* | *T. fiebrigi* | Colony resource | Honey | Not assessed | *Starmerella meliponinorum; Zygosaccharomyces mellis, Z. rouxii* and *Z. siamensis* |
| Santos et al. | 2022 | *Melipona and Tetragonisca* | *M. quadrifasciata, M. scutellaris and T. angustula* | Larval provision | Larval food isolates | Fourteen previously isolated bacterial strains; selected supernatants inhibited or enhanced antibiotic activity against | Not assessed |

| Study | Year | Bee genus | Bee species | Life stage / material | Specific niche | Bacteria reported | Fungi reported |
|---|---|---|---|---|---|---|---|
| | | | | | | susceptible and multidrug-resistant clinical bacteria | |
| Beux et al. | 2022 | *Tetragonisca* | *T. angustula* | Colony resource | Honey and pollen pots | *Bacillus; Leuconostoc; Pantoea; Staphylococcus* (culture-based isolates) | *Starmerella; Zygosaccharomyces; Candida* |
| Sarton-Lohéac et al. | 2023 | *Frieseomelitta* | *F. varia* | Adult worker | Adult hindgut | Lactobacillaceae; Bifidobacteriaceae; abundant Neisseriaceae, Orbaceae and Enterobacteriaceae; one Enterobacteriaceae ASV dominated all colonies | Not assessed |
| Sarton-Lohéac et al. | 2023 | *Melipona* | *M. seminigra, M. lateralis, M. fuliginosa, M. interrupta* | Adult worker | Adult hindgut | Lactobacillaceae; Bifidobacteriaceae; Acetobacteraceae; Streptococcaceae; *Snodgrassella* and *Gilliamella rare* | Not assessed |
| Sarton-Lohéac et al. | 2023 | *Scaptotrigona* | *S. polysticta* | Adult worker | Adult hindgut | Lactobacillaceae; Bifidobacteriaceae; abundant Neisseriaceae | Not assessed |
| Haag et al. | 2023 | *Melipona* | *M. quadrifasciata* | Adult forager | Gut-associated microbiota from whole abdomens | Bifidobacteriaceae; Lactobacillaceae; *Lactobacillus; Bombella*; unclassified Lactobacillaceae | *Candida*, especially *C. apicola*; *Starmerella*, especially S. cellae; *Zygosaccharomyces; Debaryomyces*; many low-abundance or unclassified OTUs |
| Santos et al. | 2023 | *Frieseomelitta* | *F. varia* | Larval provision | Larval food | *Lactobacillus* dominant; Acetobacteraceae abundant; *Enterococcus* and *Serratia* supported by both sequencing and cultivation; additional Firmicutes, Proteobacteria and Actinobacteria | *Cladosporium exasperatum* and *Saccharomyces cerevisiae* most abundant; low fungal richness relative to the other species |
| Santos et al. | 2023 | *Melipona* | *M. quadrifasciata* | Larval provision | Larval food | *Lactobacillus* dominant; Acetobacteraceae abundant; *Micrococcus; Pseudomonas* supported by both sequencing and cultivation; *Bacillus* detected by sequencing | *Cladosporium delicatulum* and *Penicillium citrinum* most abundant; *Didymella and Saccharomyces cerevisiae* among Melipona-shared taxa; high fungal diversity |
| Santos et al. | 2023 | *Melipona* | *M. scutellaris* | Larval provision | Larval food | *Lactobacillus* dominant; Acetobacteraceae; *Bacillus; Paenibacillus polymyxa* cultured; *Serratia marcescens* cultured; *Lysinibacillus* detected | *Cladosporium delicatulum* and *Bipolaris shoemakeri* most abundant; *Didymella* and *Saccharomyces cerevisiae* among *Melipona*-shared taxa |

| Study | Year | Bee genus | Bee species | Life stage / material | Specific niche | Bacteria reported | Fungi reported |
|---|---|---|---|---|---|---|---|
| Santos et al. | 2023 | *Tetragonisca* | *T. angustula* | Larval provision | Larval food | *Lactobacillus* dominant; Acetobacteraceae; low bacterial richness; Firmicutes and Proteobacteria | *Erysiphe quercicola* and *Saccharomyces cerevisiae* most abundant; highest fungal richness among the four species |
| de Paula et al. | 2023 | *Scaptotrigona* | *S. bipunctata, S. postica, S. tubiba* | Brood provision | Brood cells | Not assessed | *Zygosaccharomyces* spp. forming pseudohyphae; isolates closely related to the *S. depilis* symbiont and accumulating ergosterol |
| de Paula et al. | 2023 | *Melipona* | *M. quadrifasciata, M. fasciculata, M. bicolor* | Brood provision | Brood cells | Not assessed | *Zygosaccharomyces* spp. forming pseudohyphae and accumulating ergosterol |
| de Paula et al. | 2023 | *Tetragona* | *T. clavipes* | Brood provision | Brood cells | Not assessed | *Zygosaccharomyces* sp. forming abundant clumps/pseudohyphae and accumulating ergosterol |
| de Paula et al. | 2023 | *Partamona* | *P. helleri* | Brood provision | Brood cells | Not assessed | *Zygosaccharomyces* sp. forming abundant pseudohyphae and accumulating ergosterol |
| de Paula et al. | 2023 | *Melipona* | *M. quadrifasciata* | Colony resource | Honey | Not assessed | *Zygosaccharomyces osmophilus* |
| de Paula et al. | 2023 | *Tetragona* | *T. clavipes* | Colony resource | Honey | Not assessed | *Zygosaccharomyces osmophilus* |
| de Paula et al. | 2023 | *Geotrigona* | *G. mombuca* | Colony resource | Honey | Not assessed | *Zygosaccharomyces osmophilus* |
| de Paula et al. | 2023 | *Frieseomelitta* | *F. varia* | Nest material | Cerumen and honey | Not assessed | *Zygosaccharomyces siamensis* isolated from both honey and cerumen |
| de Paula et al. | 2023 | *Scaptotrigona* | *S. bipunctata* | Nest material | Brood-cell cerumen | Not assessed | *Monascus ruber* |
| de Paula et al. | 2023 | Multiple | *Tetragonisca angustula, Friesella schrottkyi, Nannotrigona testaceicornis, M. quadrifasciata, S. bipunctata* | Brood provision / nest material | Brood cells and cerumen | Not assessed | *Monascus* spp.; *Monascus ruber; Xerochrysium* sp.; *Leiothecium* sp. |
| Andrade-Velásquez et al. | 2023 | *Melipona*, *Plebeia*, *Scaptotrigona* | *M. beecheii*, *P. jatiformis*, *S. pectoralis* | Colony resource | Honey | Fructophilic LAB: *Fructilactobacillus* spp., *F. pseudoficulneus*, *F. tropaeoli* | Not assessed |

| Study | Year | Bee genus | Bee species | Life stage / material | Specific niche | Bacteria reported | Fungi reported |
|---|---|---|---|---|---|---|---|
| Roque et al. | 2024 | *Melipona* | *M. orbignyi and M. seminigra pernigra* | Adult body and gut; hive microhabitats | Body, gut and hive | Not assessed | *Starmerella apicola* and *S. etchellsii* recurrent; *Pseudozyma hubeiensis* dominant in *M. seminigra pernigra;* species-specific assemblages; Saccharomycetales dominant in metabarcoding |
| Roque et al. | 2024 | *Scaptotrigona* | *S. xanthotricha, S.* cf. *polysticta* and *S.* cf. *nigrohirta* | Adult body and gut; hive microhabitats | Body, gut and hive | Not assessed | *Starmerella etchellsii* and S. *apicola* recurrent; *Hanseniaspora opuntiae* dominant across all microhabitats of *S.* cf. *nigrohirta; Metschnikowia* spp. dominant in its gut; *S. apicola* dominant in *S.* cf. *polysticta* |
| Roque et al. | 2024 | *Scaptotrigona* | *S.* cf. *polysticta and S.* cf. *nigrohirta* | Colony resource | Honey | Not assessed | *Zygosaccharomyces rouxii* exclusive to *S.* cf. *polysticta* honey; *Hanseniaspora opuntiae* and *Wickerhamiella versatilis* among *S.* cf. *nigrohirta*-associated yeasts |
| Roque et al. | 2024 | *Scaptotrigona* | *S.* cf. *polysticta and S.* cf. *nigrohirta* | Colony resource | Stored pollen / bee bread | Not assessed | Variable yeast assemblages; no yeasts recovered from *S.* cf. *polysticta* bee bread; *Hanseniaspora opuntiae* occurred across all *S.* cf. *nigrohirta* microhabitats |
| Roque et al. | 2024 | *Tetragonisca* | *T. angustula and T. fiebrigi* | Adult body and gut; hive microhabitats | Body, gut and hive | Not assessed | *Wickerhamiella versatilis* and *Starmerella meliponinorum* across body, hive and gut of T. *angustula; Hanseniaspora opuntiae* dominant across Amazon *T. angustula* microhabitats; *Starmerella apicola, Kodamaea ohmeri* and *Rhodotorula* sp. important in *T. fiebrigi* |
| Roque et al. | 2024 | *Tetragonisca* | *T. angustula* | Colony resource | Honey | Not assessed | *Hanseniaspora opuntiae; Wickerhamiella versatilis* enriched in hive and honey |
| Roque et al. | 2024 | *Tetragonisca* | *T. angustula* | Colony resource | Stored pollen / bee bread | Not assessed | *Hanseniaspora opuntiae* present across microhabitats; additional species-specific yeast assemblage |
| Cerqueira et al. | 2024 | *Melipona* | *M. bicolor bicolor, M. mondury, M. quadrifasciata anthidioides* | Adult forager, whole surface- | Whole adult bee microbiota | *Bifidobacterium; Pectinatus; Floricoccus; Commensalibacter* and other Acetobacteraceae; *Lactobacillus; Acetilactobacillus; Ligilactobacillus.* | *Metschnikowia*/Metschnikowiaceae; Saccharomycetales including *Candida*; Cladosporiaceae; Aspergillaceae; |

| Study | Year | Bee genus | Bee species | Life stage / material | Specific niche | Bacteria reported | Fungi reported |
|---|---|---|---|---|---|---|---|
| | | | | disinfected body | | *Snodgrassella* and *Gilliamella* absent or at very low abundance. | Nectriaceae; Dissoconiaceae; *Priceomyces*/Debaryomycetaceae |
| Cerqueira et al. | 2024 | *Melipona* | *M. bicolor bicolor, M. mondury, M. quadrifasciata anthidioides* | Colony resource | Honey | *Rosenbergiella; Acinetobacter; Fructilactobacillus; Saccharibacter; Acetobacter* and other Acetobacteraceae; Alpha 2.2/*Bombella*-like strains | Saccharomycetales; *Metschnikowia*; *Candida*; *Zygosaccharomyces mellis*-like OTUs; *Starmerella cellae*-like OTUs; *Priceomyces* |
| Ramírez-Ahuja et al. | 2025 | *Frieseomelitta* | *F. nigra* | Adult worker | Adult gut | *Lactobacillus; Bifidobacterium;* variable Enterobacteriaceae-associated genera; *Weissella* not detected | Not assessed |
| Ramírez-Ahuja et al. | 2025 | *Melipona* | *M. beecheii, M. solani, M. yucatanica, M. fasciata* | Adult worker | Adult gut | *Lactobacillus* in all species; *Bifidobacterium* in most; *Weissella* variable; Enterobacteriaceae and Acetobacteraceae variable | Not assessed |
| Ramírez-Ahuja et al. | 2025 | *Plebeia* | *P. melanica, P. llorontei, P. bilineata, P. frontalis* | Adult worker | Adult gut | *Lactobacillus* in all species; *Bifidobacterium* in most; *Weissella* in three species; variable Enterobacteriaceae and Acetobacteraceae | Not assessed |
| Ramírez-Ahuja et al. | 2025 | *Scaptotrigona* | *S. pectoralis, S. mexicana, S. hellwegeri* | Adult worker | Adult gut | *Lactobacillus; Bifidobacterium; Weissella* variable; *Snodgrassella* and other Proteobacteria variable | Not assessed |
| Caesar et al. | 2025 | *Scaptotrigona* | *S. depilis* and *S. bipunctata* | Adult nurse, forager and soldier | Adult gut | Lactobacillaceae; *Floricoccus;* Bifidobacteriaceae; *Bombilactobacillus*; nurses additionally carried *Acetilactobacillus;* soldiers often dominated by *Bifidobacterium* | Variable Saccharomycetes/Saccharomycetales |
| Caesar et al. | 2025 | *Scaptotrigona* | *S. depilis* and *S. bipunctata* | Colony resource | Honey | Lactobacillaceae; Acetobacteraceae; *Acinetobacter*; high strain diversity and numerous unique taxa | Variable and rich fungal community, including Saccharomycetales |
| Caesar et al. | 2025 | *Scaptotrigona* | *S. depilis* and *S. bipunctata* | Colony resource | Stored pollen | Lactobacillaceae, especially *Acetilactobacillus/Nicoliella*-related lineages; Acetobacteraceae; *Acinetobacter pollinis* | Variable fungal community, richer and less filtered than larval food |
| Caesar et al. | 2025 | *Scaptotrigona* | *S. depilis* and *S. bipunctata* | Larval provision | Larval food | Lactobacillaceae dominated; *Acetilactobacillus*, *Nicoliella* and *Apilactobacillus* prominent; | Saccharomycetaceae/Saccharomycetales dominated, including |

| Study | Year | Bee genus | Bee species | Life stage / material | Specific niche | Bacteria reported | Fungi reported |
|---|---|---|---|---|---|---|---|
| | | | | | | *Bombilactobacillus* and *Bifidobacterium* also detected | *Zygosaccharomyces* and *Starmerella*-like yeasts |
| Caesar et al. | 2025 | *Scaptotrigona* | *S. depilis* and *S. bipunctata* | Larva | Larval body / larval microbiota | Minimal bacterial colonization; no MAG reached persistence thresholds; transient low-abundance hive strains | Predominantly Saccharomycetales/Saccharomycetaceae, including *Zygosaccharomyces* and *Starmerella*-like yeasts |
| Haag et al. | 2026 | *Melipona* | *M. quadrifasciata* and *M. mondury* | Adult worker | Adult gut | Core in both species: *Lactobacillus, Bifidobacterium, Apilactobacillus, Bombella* and *Floricoccus;* Snodgrassella usually rare; low-prevalence spillover taxa from *Apis* | Not assessed |
| Santini et al. | 2026 | *Melipona* | *M. quadrifasciata anthidioides* | Adult forager | Whole gut and gut regions | Core-like *Bifidobacterium*, *Lactobacillus, Apilactobacillus, Bombella* and *Floricoccus; Neokomagataeae* in crop; *Gilliamella, Snodgrassella* and *Bombilactobacillus* generally absent | Not assessed |
| Xolalpa-Aroche et al. | 2026 | *Melipona* | *M. beecheii* | Colony resource | Honey | *Acetilactobacillus*-like; *Nicoliella*-like Lactobacillaceae; genome-resolved metagenomics identified four novel Lactobacillaceae species dominating pot honey | Not assessed |
| Xolalpa-Aroche et al. | 2026 | *Scaptotrigona* | *S. mexicana* | Colony resource | Honey | *Acetilactobacillus*-like; *Nicoliella*-like Lactobacillaceae; genome-resolved metagenomics identified four novel Lactobacillaceae species dominating pot honey | Not assessed |
